# Properties of Structural Lightweight Aggregate Concrete Based on Sintered Fly Ash and Modified with Exfoliated Vermiculite

**DOI:** 10.3390/ma14205922

**Published:** 2021-10-09

**Authors:** Patrycja Przychodzień, Jacek Katzer

**Affiliations:** Department of Building Engineering, Faculty of Geoengineering, Institute of Geodesy and Civil Engineering, University of Warmia and Mazury in Olsztyn, 10-724 Olsztyn, Poland; jacek.katzer@uwm.edu.pl

**Keywords:** lightweight aggregate, sintered fly ash, exfoliated vermiculite, durability

## Abstract

Despite the undoubted advantages of using lightweight concrete, its actual use for structural elements is still relatively small in comparison to ordinary concrete. One of the reasons is the wide range of densities and properties of lightweight aggregates available on the market. As a part of the research, properties of concrete based on sintered fly ash were determined. The ash, due to its relatively high density is suitable to be used as a filler for structural concretes. Concrete was based on a mixture of sintered fly ash and exfoliated vermiculite aggregate also tested. The purpose of the research was to determine the possibility of using sintered fly ash as alternative aggregate in structural concrete and the impact of sintered fly ash lightweight aggregate on its physical, mechanical and durability properties. Conducted tests were executed according to European and Polish standards. Created concretes were characterized by compressive strength and tensile strength ranging from 20.3 MPa to 54.2 MPa and from 2.4 MPa to 3.8 MPa, respectively. The lightest of created concretes reached the apparent density of 1378 kg/m^3^.

## 1. Introduction

Concrete structures are ubiquitous in today’s world. At the moment, concrete, as the structural material, does not have a worthy replacement. Due to large-scale production of concrete, the construction industry significantly impoverishes the deposits of natural raw materials, including aggregates which constitute majority of concrete volume. Moreover, in an increasingly environmentally conscious world, concrete production is blamed for a negative impact on the environment and subsequently climate change [1,2]. The 21st century presents the construction sector with a challenge in terms of sustainable development. The “circular economy” is emphasized by the European Union. The use of secondary raw materials before natural ones is highly encouraged in the EU. Natural aggregate can be successfully replaced by waste materials [3,4,5]. An example of such material is sintered fly ash (SFA). Fly ash is one of the byproducts of fossil fuels (especially hard coal and lignite) combustion. Poland is the European leader in the production of energy from coal-fired power plants [6]. In 2019, almost 2 million tons of fly ash was produced and about 26 million tons have already been accumulated in landfills [7].

There are many reasons to increase the amount of Coal Combustion Products development. Firstly, the landfill area can be used differently. Secondly, selling the by-products as raw material can generate financial gains, or at least offset processing and storage costs. Thirdly, it should be remembered that ash is a waste and if not properly disposed it may pose a threat in the form of possible contamination of soil, air and water. It also carries significant risk of spontaneous combustion [8]. The production of lightweight aggregates from SFA is probably the most promising method of its utilization. The use of industrial waste as a building material is a sustainable disposal practice preserving natural resources for future generations.

SFA is classified as lightweight aggregate. Concrete based on SFA, in addition to being a pro-ecological material, is characterized by reduced weight and higher thermal, acoustic and fire resistance in comparison to ordinary concrete. The specific properties of lightweight concretes are utilized in the construction of tall buildings, long span bridges, buildings erected in seismic zones [9] and in all types of structures where weight reduction can reflect overall savings.

While the use of lightweight concrete is constantly increasing due to its practical, economic and environmental advantages, it is still a contentious technological and designing challenge for many engineers, architects and contractors. The majority of this “reserved” attitude is caused by bad experiences with low-quality lightweight aggregates for less demanding applications (e.g., insulating concretes) in the past decades. Light aggregates, unlike natural aggregates, have a very wide range of density (from 400 to 2000 kg/m^3^) and strength [4]. SFA is light aggregate on the basis of which lightweight structural concrete can be designed. The density of SFA concrete ranges from 1450 kg/m^3^ [9]. The aggregate that allows obtaining the lowest density of lightweight concrete (from 300 to 800 kg/m^3^) is vermiculite. This is also the aggregate which can be used only for production of insulation concrete [9]. Therefore, the research program was focused on testing how two very different light aggregates work together in one concrete. The key novelty of the study is associated with using simultaneously two very different types of lightweight aggregates. By mixing them together in different proportions one can achieve very wide range of mechanical and physical properties. Such an approach is novel and offer new type of lightweight concretes.

## 2. Materials and Concrete Mix Design

### 2.1. Materials

As a key ingredient SFA aggregate produced in Poland by the LSA was used. The material base for the production of this LSA aggregate is fly ash from the combustion of hard coal from the Białystok Power Station. The production of LSA aggregate utilizes around 60,000 tons of fly ash per year from the heap. The size of the heap was assessed at approximately 1.2 million tons in 2016. The processing technology is based on high-temperature (1000–1200 °C) sintering in the rotary kiln. Fly ash does not swell during sintering. The result of this process is a coating that is much less porous than the internal structure of the aggregate. The achieved material is less absorbent, has higher mechanical strength but is much heavier than other light aggregates, such as pumice or vermiculite [10,11,12].

**Figure 1:**
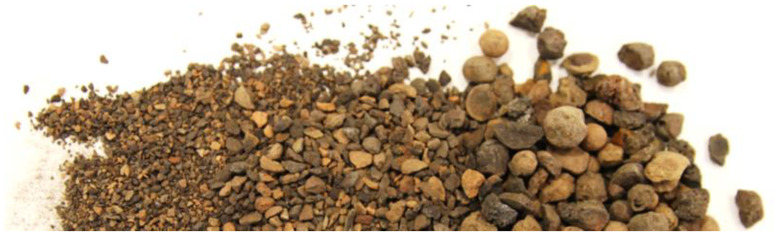
SFA aggregate divided by fractions (from left: 0/2 mm; 1/4 mm and 4/8 mm).

**Figure 2:**
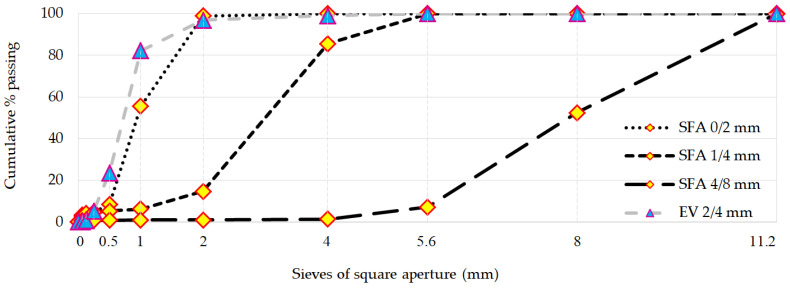
Grading curves of SFA and EV aggregates.

In experimental studies, three fractions of aggregates 0/2 mm; 1/4 mm (achieved by crushing larger particles) and 4/8 mm (as crushed aggregate in about 90% [13]), were used. In Figure 1 pictures of all three fractions are presented. Sieve analysis was carried out according to EN 933-1:2012 [14]. The grading curve is shown in Figure 2. Key properties of both light aggregates used in the study are summarized in Table 1. The apparent density was determined utilizing the method described in the Polish standard PN-B-06714 -06:1976 [15]. Water absorption, bulk density and water content were determined according to EN 1097-6:2013 [16], EN 1097-3:1998 [17] and EN 1097-5:2008 [18], respectively.

**Figure 3:**
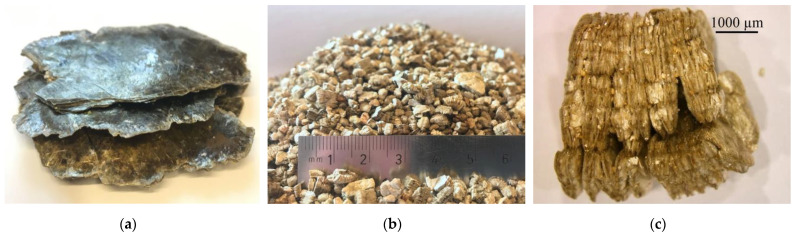
Vermiculite (**a**); raw vermiculite; (**b**) exfoliated vermiculite fraction 2/4 mm; (**c**) exfoliated vermiculite pellet.

Exfoliated vermiculite (EV) was used as the second light aggregate. Vermiculite is a naturally occurring mineral from the group of hydrous layer aluminosilicates. It is widespread around the world together with other clay minerals. Significant deposits of raw vermiculite are located in Australia, China, the USA and South Africa [19,20,21]. The highest volume of vermiculite is produced by South Africa, followed by the USA, Brazil, Zimbabwe and Russia. For over 20 years, the annual worldwide production of vermiculite has been equal to circa 500,000 tons. Coarse-grained raw materials (particles larger than 2 mm) are the most desirable and there is a significant shortage of them [19]. The production of fine-grained vermiculite exceeds the demand; therefore, additional ways of using them are required [19,20,21]. The process of exfoliation of raw vermiculite takes place at high temperatures ranging from 400 to 1000 °C. After exfoliation, the grains can increase their volume up to 30 times [22,23]. For the needs of the current research program the material was supplied by Vermiculite Poland. In Figure 3 vermiculite, raw vermiculite, exfoliated vermiculite fraction 2/4 mm and exfoliated vermiculite pellet are presented.

Particle size analysis of EV (Figure 2) indicates the high amount of 0/2 mm fraction. One should keep in mind that traditional sieve analysis is not reliable in case of EV. Delicate EV particles are crushed during the sieving process. The aggregate grading curve in question was created after mechanical fragmentation of vermiculite (fraction 2/4 mm according to manufacturer’s declaration).

The apparent density of lightweight aggregates varies greatly (from 400 to 2000 kg/m^3^). This phenomenon is caused by significant porosity of particles (from 40% to 80%). The porosity of lightweight aggregates usually increases with the size of the aggregate; therefore, coarse fractions are characterized by lower apparent density than fine fractions. SFA is characterized by high density as for light aggregate. However, its density is still within the limits of lightweight aggregate characteristics. It constitutes around 65% of the density of ordinary aggregates (2400–2600 kg/m^3^). In case of EV the apparent density was not determined. The bulk density of EV was more than five times lower than bulk density of SFA of similar diameter (1/4 mm).

In this study, crushed coarse aggregate was used. Therefore, it has lower strength and increased water absorption in comparison to non-crushed (rounded) particles. Water absorption of crushed and non-crushed particles of the same light aggregate was determined by Zhang and Gjørv [24]. Their study revealed that crushed light aggregate can be even 50% more absorbent than non-crushed. They also pointed out that lightweight aggregates absorb most of the water within the first 2 min, while the remaining water is absorbed at much slower pace.

A mix of Portland cement 42.5 R (CEMEX, Chełm, Poland) and an active additive of type II zeolite (ZeoBau 50, ASTRA, Straszyn, Poland) was used as a binder [25,26]. Zeolite is a natural rock from the group of aluminosilicate minerals with different chemical composition, properties and crystal form. It is common all over the world with various chemical compositions. The largest producers of zeolite are China, South Korea, Slovakia and New Zealand [21]. In Poland, zeolite is provided by ASTRA [27] and this product was used in the research. Zeolite was used to partially substitute cement. The main aim of using zeolite was to fulfill requirements of cement consumption limits. Moreover, zeolite acts as a stabilizer in concrete mixtures. It prevents the segregation of components, which in lightweight concrete is a frequently occurring problem [27]. A superplasticizer admixture was used to create all concrete mixes. Namely ViscoCrete 41 RS (by Sika, Sika Poland, Warsaw, Poland) was utilized. This is a second-generation comb-type water reducer based on polycarboxylate (PCE). It also contains stabilizing polymers, which hinder the segregation of concrete mix ingredients [28]. Last but not least, ordinary tap water was used as mixing water.

### 2.2. Concrete Mix Design

**Figure 4:**
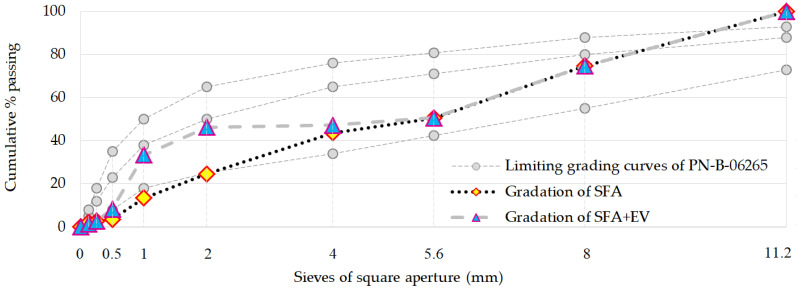
Grading curves of SFA and SFA + EV aggregate mixes.

Firstly, the good aggregate grading (through the experimental method) was achieved using three fractions of SFA. The process is based on mixing different aggregate fractions until the maximum compacted bulk density is achieved. The tests showed that the proportions (by volume) for the optimal grading are as follows: SFA 0/2 mm—20.51%; SFA 1/4 mm—26.23%; SFA 4/8 mm—53.25%. This was the first aggregate mix used in the research program. In the second aggregate mix, the SFA 1/4 mm was replaced by EV 2/4 mm. The changes were made by volume. The apparent density of EV is unknown. Therefore, the mass demand for new aggregate was estimated on the basis of the results of the research on the bulk density and the percentage content of the SFA 1/4 mm fraction of SFA in the first aggregate mix. For both SFA and SFA + EV aggregate mixes, sieve analysis was conducted. Grading curves are shown in Figure 4 together with limiting grading curves for ordinary concrete required by Polish standards PN-B-06265 (Annex P) [29]. Both aggregate mixes can be considered as coarse-grained.

The concrete mix was designed using the iteration method. The desired consistency of the fresh mix was S2 [30]. Porous aggregate absorbs some of the liquid components of the fresh concrete mix. To eliminate this phenomenon, it was decided to use pre-moistened aggregates. The aggregates were exposed to 30-min absorbency of water as suggested by Zhang and Gjørv [24]. Due to 30–60 min exposure to water absorbency, the dynamic of absorption is relatively stabilized [4]. In this was the amount of water needed for moistening the aggregate was assessed as suggested by Nadesan and Dinakar [31].

The mix of cement and zeolite (in proportion 85% and 15%) was used as a binder. A pan type concrete mixer was utilized for the creation of the concrete mixes. The procedure of concrete mix preparation was as follows. Firstly, aggregate and water for aggregate were placed in the mixer. Secondly, the water and aggregate were pre-mixed and left for 15 min. Thirdly, cement, zeolite and 2/3 of mixing water were added to the mixer. During mixing the superplasticizer with the last part of mixing water was slowly added to the mixer. The recipes of both SFA and SFA + EV are given in Table 2. The consistency and density of fresh mixes were tested according to EN 12350-2:2019 [30], EN 12350-6:2019 [32], respectively. The slump test results for SFA and SFA + EV mixes were equal to 45 mm and 35 mm, respectively. These results reflect the results achieved by Terzić et al. [33] who described in details the influence of EV on the consistency of fresh concrete mix.

The density of fresh SFA and SFA + EV fresh concrete mixes was equal to 1790 kg/m^3^ and 1738 kg/m^3^, respectively. It can be observed that density decreased by about 50 kg/m^3^ due to the use of vermiculite. The volume of the components in their initial state (before combining) was the same. During mixing, the brittle grains of vermiculite crumbled, resulting in the reduction of the volume of the created fresh SFA + EV concrete mix (by about 7% in comparison to SFA fresh concrete mix).

### 2.3. Specimens and Testing

The 100 mm cube specimens were prepared for water absorption, freeze-thaw resistance (Uni-Mors, Grodzisk Mazowiecki, Poland), compressive (Controls, Milan, Italy) and tensile strength tests (Controls, Milan, Italy). The 150 mm cube specimens were used to determine water permeability (MULTISERW-Morek, Brzeźnica, Poland). Both types of cubes were utilized to establish density of hardened concrete at various levels of water absorption. The specimens were cast in three layers in plastic molds and compacted on a vibration table. After casting and vibrating the molds were cover with plastic foil to prevent water evaporation. Specimens were demolded after 48 h of curing. Earlier demolding causes defects, cracks and other damages to the concretes in question. Specimens were kept in a curing tank with a relative humidity of 95% until the specified age of testing. All tests were conducted using minimum three specimens.

## 3. Results and Discussion

### 3.1. Density

**Figure 5:**
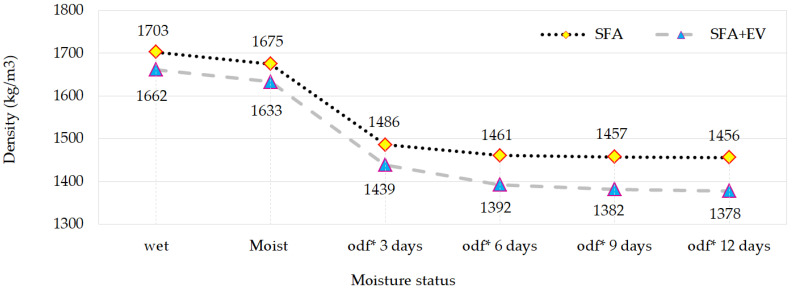
Density of SFA and SFA + EV concretes in different saturation states (odf*—oven dry for).

Density of SFA and SFA + EV concretes was determined in different saturation states. The following saturation states were considered: wet, moist (relative humidity ±95%) and after 3, 6, 9 and 12 days of drying at 105 °C. Density in the different saturation states is presented in Figure 5.

The density of lightweight concrete is its key property in combination with the strength class. It is thanks to the reduced density that the weight of a structure with the same load-bearing capacity can be reduced by 20 or even 40%. The density of the concrete mix depends on the density and proportion of its components, the air content and (in the case of lightweight concrete), on the initial moisture level of porous aggregates. The change in the density of the built-in concrete is caused by a change in the internal saturation, which results from the prevailing climatic conditions and the internal structure. This means that the concrete in the structure is not dry, but is in equilibrium with the relative humidity of the environment, which will vary depending on the exposure, geographic location and season of the year. Therefore, the properties of concrete should be related to the actual state, taking into account the influence of humidity, which, as a rule, can increase the density of concrete by 50–200 kg/m^3^ [4,34].

In the case of lightweight concrete, the loss of moisture through drying is a longer process in comparison to an ordinary concrete. It is related to the water in the aggregate pores. The oven-dry state of the specimens (drying to a constant mass) was achieved after 12 days of keeping specimens in the temperature of ±105 °C. The greatest dynamics of weight loss occurs during the first three days of drying and then the process slows down significantly. The study showed that even concretes saturated with water can be classified as lightweight concretes with density below 2000 kg/m^3^. The vermiculite aggregate absorbs and stores more water than SFA. The difference in density between SFA and SFA + EV concretes in the wet state is around 40 kg/m^3^ and in the dry state it is around 80 kg/m^3^.

### 3.2. Water Absorption

**Figure 6:**
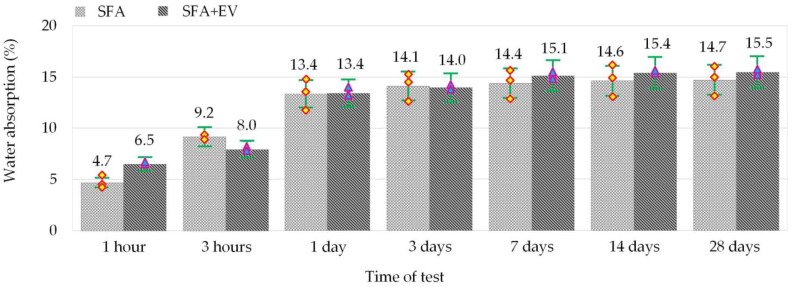
Water absorption of SFA and SFA + EV concretes.

Such properties of concrete as water absorption and watertightness largely depend on the humidity of the specimens [35,36]. Therefore, after the curing, the specimens were dried in an oven at +105 °C for 72 h and left in air-dry conditions until constant mass was achieved. Dry specimens were used to test the water absorption phenomenon. The specimens were immersed in water for various periods of time varying from 1 h to 28 days. The absorption results achieved in this way are presented in Figure 6.

Dynamic increase in water absorption of the specimens was observed during the first 24 h. The next days are a period of stabilization, during which the increase of water absorption is within the limits of less from 0.01% to 0.39% per day. The 24 h water absorption constitutes 91% and 87% of full water absorption of SFA and SFA + EV concretes respectively.

Lightweight structural concrete is characterized by water absorption from 5 to 25%. This considerable variation is caused by properties of used lightweight aggregates. Domagała [37] demonstrated that pre-saturating aggregate and w/c are the most important factors influencing the water absorption of concretes with fly ash aggregates.

SFA + EV concrete achieved slightly higher (by 0.74%) final water absorption than SFA concrete. SFA + EV concrete absorbed more water than SFA concrete in the first hour of the test. This phenomenon is caused by the presence of the vermiculite material in the cement matrix which is characterized by high sorptivity. The porosity of the cement matrix and pores between the layers of vermiculite were confirmed by numerous researchers to influence the water absorption [22,23,38]. For concrete based only on vermiculite (65% volume of concrete) and natural sand water, absorption can even reach 40% [23].

The test method and the water absorption limits are not specified in the concrete standard EN 206 [39]. Therefore, a reference to the Polish standard PN-91/B-06263 [40] was made. According to the standard the water absorption of concrete which under operating conditions is exposed to elements should not exceed 20%. Both concretes in question fulfilled this condition.

That is a satisfying result which was obtained thanks to complete initial saturation of the aggregate. As a consequence of water absorption by the aggregate during curing, the w/c ratio decreased and the aggregate particle structure was sealed with cement paste.

### 3.3. Water Permeability

As proved by many researchers [36,37,41], the permeability of lightweight concretes may be lower than that of ordinary concretes, despite their higher water absorption. The basic properties determining the permeability of concrete is the quality of the transition zone between aggregate and cement matrix and matrix structure. This is more important than the porosity of the aggregates. Proper curing and prevention of cracks are therefore essential [35,41].

In previous tests conducted by other research teams [35,37] attention has been paid to a very important aspect of the initial pre-moisture of the material. The research by Domagała [37] showed that obtaining concrete with relatively low permeability is possible even when using aggregates with quite high water absorption of about 19% and w/c of 0.55. Although the EN 12390-8 [42] standard does not define how the specimens should be prepared for testing, the tests [35,36] focused on the moisture condition of the specimens before tests. This has an impact on the final results. When the pore system is unsaturated, capillary absorption dominates. When they are saturated, there is a movement of water, but only when high pressure occurs. Therefore, if the specimens are oven dry, permeability may be higher than that of identical specimens tested at natural humidity. Kockal and Ozturan [36] mentioned that the higher permeability of dry samples may also be caused by the appearance of microcracks due to high temperatures during drying. In their tests, the permeability of such specimens was twice as high as for specimens that were not subjected to high temperatures before the test. Azad et al. [43] proved that the presence of vermiculite may reduce permeability. This phenomenon is caused by the high water absorption of vermiculite which stores water but does not have a large capacity to transport it.

**Figure 7:**
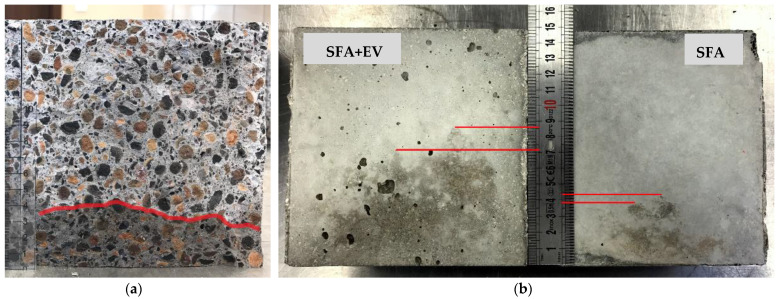
Depth of penetration of water under pressure: (**a**) cross-section of a specimens of SFA concrete; (**b**) surface of SFA + EV concrete and SFA concrete specimens after the test.

During the current research program concrete was made using aggregate in a pre-wetted state. The depth of penetration of water under pressure was determined with apparatus generating water pressure from the bottom of specimens. The testing procedure consisted of water pressure from 0.4 to 0.8 MPa which was kept for 100 h. Cement paste was removed (by grinding) from the surface of a specimen which was going to be exposed to water pressure (a circular area with the diameter of 10 cm). After the test, specimens were split and the cross-section was examined and the penetration range was measured (Figure 7). The average penetration depths of 3.7 cm and 7.6 cm for SFA concrete and SFA + EV concrete were noted respectively.

The current European standard [40] does not define the depth range when concrete can be considered waterproof or not. The German DIN 1045-2 [44], national addendum to Standard EN 206 [39], can be helpful in determining waterproofnes based on penetration depth. As a criterion, it specifies:

− Depth ≤50 mm—high resistance against weak chemical substances.
− Depth ≤30 mm—high resistance against strong chemical substances.

Based on the above conditions, the SFA concrete showed adequate resistance to exposure to weak chemicals substances. Concrete SFA + EV did not meet any of the requirements. The research did not confirm the effect of vermiculite presented by Azad et al. [43]. Vermiculite greatly facilitated the transport of water through the concrete matrix.

### 3.4. Compressive Strength

**Figure 8:**
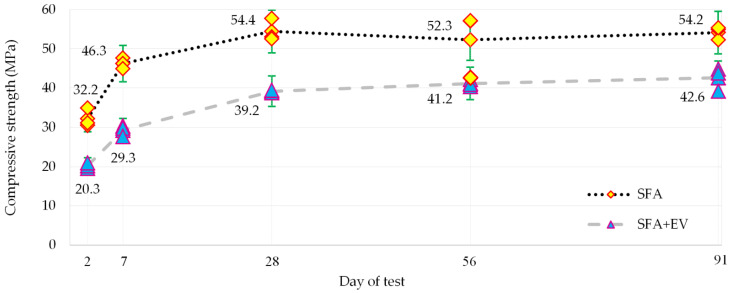
Compressive strength.

Compressive strength of hardened concrete specimens was determined by the destructive method in accordance with EN 12390-3 [45]. The specimens were tested after 2, 7, 28, 56 and 90 days of curing. Constant loading rate of 0.75 MPa/s was applied. The compressive strength was calculated based on the obtained maximum load at failure and the cross-sectional area on which the compressive force acted. Results were shown with in Figure 8.

EV as a partial replacement for aggregate caused a reduction in compressive strength in comparison to SFA. This phenomenon is caused by the properties of the vermiculite itself which in turn results in decrease in concrete density and an increase in porosity [22,46]. Compressive strength of SFA + EV concrete reached from 21 to 37% (28% for the 28th day) of strength of SFA concrete.

The concretes showed high early strength. The 2-day compressive strength, which was determined on the day of demolding of the specimens, constituted around 59 and 48% of the maximum strength achieved by SFA and SFA + EV concretes respectively. In both cases, after 28 days of curing the dynamic development of endurance ends. However, in the case of SFA + EV, the increase in the following days is much lower but is still gradually progressing. SFA strength results after 56 and 91 days of curing are inconclusive. The decrease of average strength of SFA concrete after 56 days is caused by significant dispersion of single results.

### 3.5. Tensile Strength

**Figure 9:**
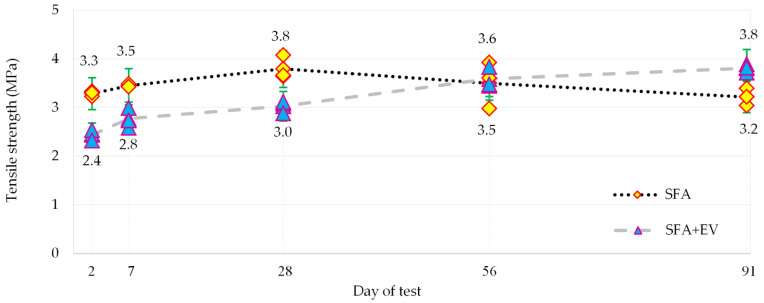
Tensile strength.

The tensile strength was tested with the help of the splitting method according to EN 12390-6 [47]. Constant loading rate of 1500 N/s was applied during the test. The achieved results of tensile strength test are presented in Figure 9.

SFA concrete test results until the 28th day of curing increase. The 2-day strength reached 87% of the 28-day strength. After 28 days of curing, results start to be scattered; thus, the average value decreases. The 91-day strength is almost equal to 2-day strength.

The strength of SFA + EV concrete increased less dynamically than SFA concrete during early days of curing. Despite lower early strength (after 2 and 7 days) and lower strength after 28 days of curing, the strength in late age was higher than that of SFA concrete. The results of SFA + EV concrete show a continuous increase in strength over time. SFA + EV concrete demonstrates an increase in late strength in comparison to SFA concrete.

### 3.6. Freeze/Thaw Resistance

The freeze/thaw resistance of lightweight concrete is a very complicated and multifaceted issue. It depends largely on material factors, but in structural lightweight concrete even more on technological factors. The most important of them is process of pre-wetting of aggregates. The influence of each degree of saturation (dry, pre-wetted, saturated) was tested by other researchers [37,48]. The best freeze/thaw resistance is reached using dry aggregates. It is possible to obtain a lightweight concrete characterized by freeze-thaw resistance comparable with an ordinary concrete [35]. The larger the pre-wetting degree, the lower the resistance to freeze/thaw cycles and the greater mass loss [37,48,49]. The research by Domagała [37] showed that the pre-soaked aggregate made the concrete unable to withstand even 30 freezing/thawing cycles.

**Figure 10:**
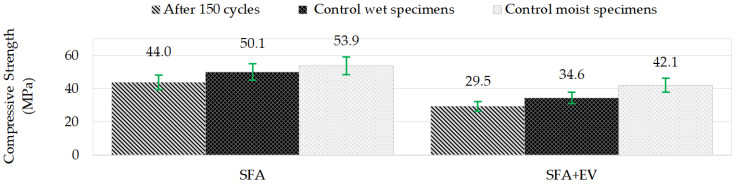
Compressive strength before and after freeze/thaw cycles.

**Figure 11:**
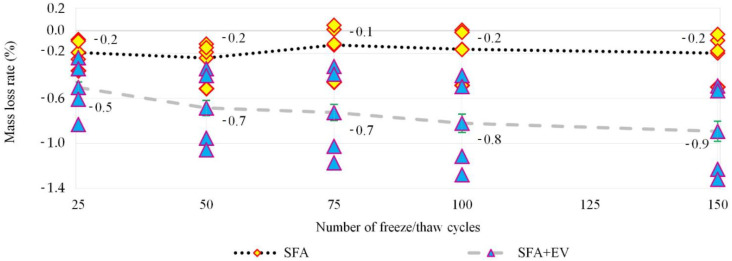
Mass loss of specimens during freeze/thaw cycles.

During the current research program the used aggregate was pre-wetted at the level of 13% and 16% for fine and coarse aggregate, respectively. The freezing/thawing cycles test was performed in a climatic chamber. 150 freezing/thawing cycles were executed. One cycle consisted of 4 h of freezing in the air at −15 °C and 4 h of thawing in water at +15 °C. Four specimens were used for each test. As control specimens, 3 cubes kept in water and 3 cubes kept in relative humidity of ±95% were prepared. The strength of specimens after freeze/thaw cycles and strength of control specimens are shown in Figure 10. Specimens’ mass loss is shown in Figure 11.

The European standard EN 206 [39] does not define the criteria for assessing resistance to freezing/thawing cycles; thus, the criteria presented in PN-B-06265 [29] (national supplement to EN 206 [39]) were applied. The criteria are as follows: specimens have to be free of cracks, the decrease in compressive strength cannot be more than 20% in comparison to the control specimens and mass loss cannot be larger than 5%. Comparing the results of compressive strength after 150 cycles of freezing/thawing with the control specimens in wet condition, decreases of 12.2% and 14.9% were noted for SFA concrete and SFA + EV concrete, respectively. Both concretes can be assigned to frost resistance class F150 [29]. If we take into account the results after 150 cycles of moist samples, decrease of compressive strength would be 18.3% and 29.9% for SFA concrete and SFA + EV concrete, respectively. The decrease in concrete compressive strength due to vermiculite addition (SFA concrete and SFA + EV concrete) equals 21.9; 30.9 and 33% for specimens that are moist, wet and after frost resistance testing, respectively. This means that long-term direct contact with water has a significant destructive effect on the mechanical properties of the tested lightweight concretes. Due to prolonged contact with water, numerous pores inside the material, which had alleviated the stresses caused by freezing, filled with water and ceased to function as a reserve volume for water expansion. The content of vermiculite exacerbates this problem.

**Figure 12:**
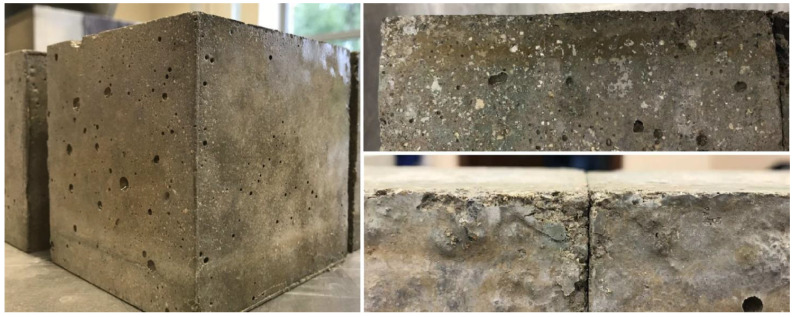
Surfaces of specimens after freezing/thawing cycles.

The recorded weight losses for single specimens were very diversified. In this case, the SFA + EV concrete showed a significant dispersion of the results. The average level of mass loss was 0.24 and 0.89% for SFA concrete and SFA + EV concrete, respectively. Freezing/thawing cycles influenced the loss of strength to a greater extent than the loss of mass. Surface flaking of specimens was also observed as shown in the Figure 12.

### 3.7. Microstructure

**Figure 13:**
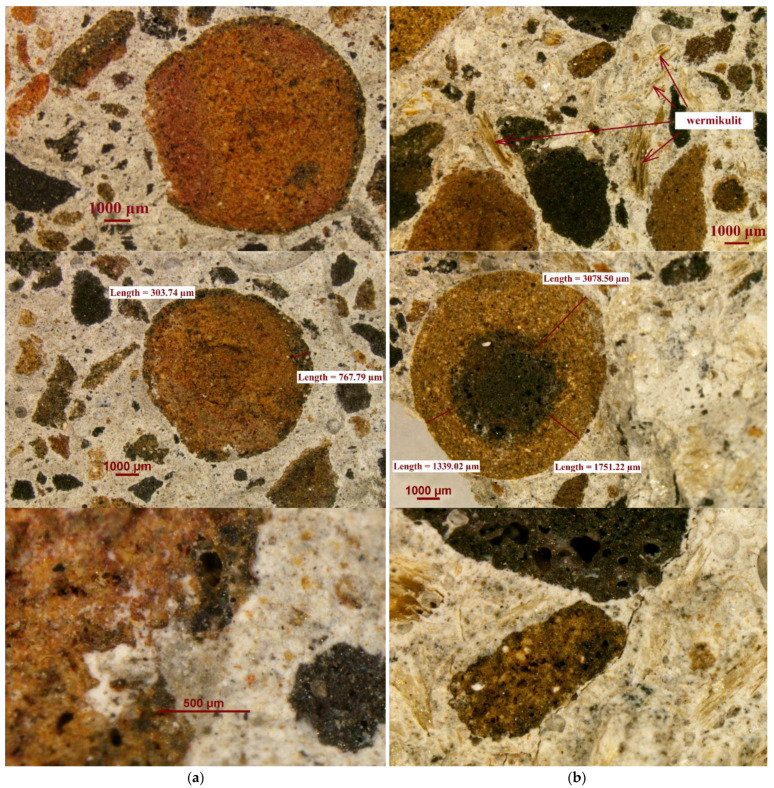
Microscopic photos of (**a**) SFA concrete and (**b**) SFA + EV concrete.

The microstructural characteristics of concretes in question were analysed using a Nikon SMZ 1500 stereoscopic microscope. No segregation of the components was observed. Despite the large differences in density between the materials used, they were homogenously distributed in the concrete volume. The microscopic photos (Figure 13) also confirmed that the destruction of concrete crack formation was going through the aggregate particles. In ordinary concrete, aggregate particles are mostly intact and the destruction runs along the cement slurry and the aggregate–slurry contact zone.

The analysis of the photos taken showed that:

A clear sinter line is visible in the aggregates of the largest fraction. The microstructure of the interior is full of pores of various sizes and shapes. The coating produced by sintering is an element of very different thickness and due to the fact that it is less porous; it can limit the penetration of water into the aggregates, if the aggregate particles are intact.

The surface of the lightweight aggregate is rough, which increases the aggregate/ slurry interface. The interfacial contact zone shows no delamination or damage, it is continuous, tight and without large pores. It indicates a strong bond between the matrix and the aggregate.

Cement hydration products penetrate the structure of the open porosity of the aggregate particles. Closed pores remain without hydration products.

### 3.8. Limitations of Cconducted Research

The conducted research program is limited by the types of tested mixes and utilized technological approaches. The research should be continued taking into account the significant material and technological influence on the properties of lightweight concretes in question. Special interest should be focused on variability of the results which was noticed during current research program. Very large population of specimens would be desired enabling full statistical analysis. Both used lightweight aggregates were sourced from one region. The same tests should be repeated using SFA and EV aggregates provided by other producers from other geographic regions. Possible further research programs should include tests of thermal, fire-retardant and acoustic properties of the concretes in question. Concrete mixes with different volumes of SFA and EV aggregates should be also tested.

## 4. Conclusions

SFA aggregate is a good alternative to ordinary aggregates. Concrete based on SFA can be successfully used in elements with load-bearing functions. It is a pro-ecological product that perfectly fits the idea of sustainable development. After the aggregate with insulating properties is incorporated into the crumb pile, the concrete can still perform the load-bearing functions. After the tests of both types of concrete, the following conclusions can be drawn:

The term "fraction" in reference to vermiculite aggregate is unreliable. Fine grains of vermiculite disintegrate (crumble, delaminate) during sieve analysis, mixing, etc., which is a problem when trying to determine the grain size and select a tight aggregate composition.

Despite significant differences in the density of components in the concrete based SFA + EV aggregate, no segregation was observed. The fresh mix showed adequate homogeneity and viscosity.

Partial replacement of the SFA aggregate by much lighter EV aggregate reduced the density of the mixture by about 50 kg/m^3^.

Adding vermiculite aggregate to the concrete mix resulted in obtaining a greater water demand. Consequently, with the same amount of water, a lower consistency class of the concrete mix was obtained.

After combining the components of the mixture, the vermiculite is crushed and partially compressed, so despite the use of the same volume of individual components, the volume of the finished mixture is reduced by about 7% compared to the mixture based on SFA aggregate only. As a result, the consumption of other ingredients per 1 m^3^ of concrete is higher.

The curing conditions of lightweight concretes have a huge impact on their mechanical and durability properties. Keeping the specimens in 95% relative humidity is preferable.

The maximum water absorption of the specimens was 15%.

With SFA aggregates, without any special technological measures, it is possible to achieve concrete of the LC 40/44 class and higher from mixed aggregates SFA + EV concrete of the LC 30/33 class and higher.

The addition of vermiculite may increase the tensile strength in later maturation periods (>28 days). It affects the plastic behaviour of concrete. This should be confirmed in further studies.

The most important disadvantage of using vermiculite is the reduction of compressive strength.

The presence of vermiculite makes the concrete more homogeneous in most cases.

Taking into account the large material and technological influence on the properties of lightweight concretes, a significant variability of the results was observed in the tests, even in a batch of samples made of the same mix. Therefore, the tests should be carried out on a larger population of samples (three samples were used for each test; not less than nine is recommended), trying to make the technological process of making subsequent samples as regular as possible.

The further part of the research should include thermal, fire-retardant and acoustic properties, as well as the next higher and lower volume of vermiculite content. One should also focus on the influence of technological conditions of execution and preparation.

The future application of the concretes in question covers a wide range of construction industry areas from thermal insulating panels, light secondary structural elements and acoustic insulation elements. Elements resistant to vibrations and absorbing energy of impacts are also possible to achieve after additional research.

## Figures and Tables

**Table 1:** Properties of SFA and EV aggregates.

Aggregates	Apparent Density (kg/m^3^)	Loose Bulk Density (kg/m^3^)	Bulk Density (kg/m^3^)	Humidity (%)	Water Absorption (wt.%)			
					After 2 min	After 30 min	After 24 h	Total
SFA 0/2 mm	1730	737	861	0.11	-	-	-	-
SFA 1/4 mm	1680	705	808	0.15	13.1	13.4	18.6	21.1
SFA 4/8 mm	1680	715	796	0.18	14.6	16.5	18.3	21.7
EV 2/4 mm	-	147	151	-	-	-	-	-

**Table 2:** Composition of concretes for 1 m^3^ (kg/m^3^).

Type of Lightweight Aggregate Concrete	Aggregates				Binder		Mixing Water	Water for Aggregate	Water-Reducing Admixture
	SFA 0/2 mm	SFA 1/4 mm	SFA 4/8 mm	EV	Cement	Zeolite			
SFA	192.5	231.1	462.1	-	480.8	84.8	212.5	132.9	3.9
SFA + EV	207.5	-	498.0	46.63	518.1	91.4	229.0	143.2	4.1

## Data Availability

The data presented in this study, are available on request from the corresponding author.
